# Structural Study of (Hydroxypropyl)Methyl Cellulose Microemulsion-Based Gels Used for Biocompatible Encapsulations

**DOI:** 10.3390/nano10112204

**Published:** 2020-11-05

**Authors:** Evdokia Vassiliadi, Evgenia Mitsou, Spyridon Avramiotis, Christos L. Chochos, Franz Pirolt, Martin Medebach, Otto Glatter, Aristotelis Xenakis, Maria Zoumpanioti

**Affiliations:** 1 Institute of Chemical Biology, National Hellenic Research Foundation, 48, Vassileos Constantinou Ave., 11635 Athens, Greece; evassiliadi@eie.gr (E.V.); emitsou@eie.gr (E.M.); spavramiotis@yahoo.com (S.A.); chochos@eie.gr (C.L.C.); arisx@eie.gr (A.X.); 2 Laboratory of Biotechnology, Department of Biological Applications and Technologies, University of Ioannina, 45110 Ioannina, Greece; 3 Anton Paar GmbH, Anton Paar Straße 20, 8054 Graz, Austria; franz.pirolt@anton-paar.com (F.P.); martin.medebach@anton-paar.com (M.M.); 4 Institute of Inorganic Chemistry, Graz University of Technology, Stremayrgasse 9, 8010 Graz, Austria; otto.glatter@uni-graz.at

**Keywords:** scanning electron microscopy (SEM), small angle X-ray scattering (SAXS), electron paramagnetic resonance (EPR), (hydroxypropyl)methyl cellulose (HPMC), lipase

## Abstract

(Hydroxypropyl)methyl cellulose (HPMC) can be used to form gels integrating a w/o microemulsion. The formulation in which a microemulsion is mixed with a hydrated HPMC matrix has been successfully used as a carrier of biocompatible ingredients. However, little is known about the structure of these systems. To elucidate this, scanning electron microscopy was used to examine the morphology and the bulk of the microemulsion-based gels (MBGs) and small-angle X-ray scattering to clarify the structure and detect any residual reverse micelles after microemulsion incorporation in the gel. Electron paramagnetic resonance spectroscopy was applied using spin probes to investigate the polar and non-polar areas of the gel. Furthermore, the enzyme-labelling technique was followed to investigate the location of an enzyme in the matrix. A structural model for HPMC matrix is proposed according to which, although a w/o microemulsion is essential to form the final gel, no microemulsion droplets can be detected after incorporation in the gel. Channels are formed by the organic solvent (oil), which are coated by surfactant molecules and a water layer in which the enzyme can be hosted.

## 1. Introduction

Gels are defined as three-dimensional macromolecule networks swollen by large amounts of solvent and are divided in different categories according to their ingredients and formation procedure. Their properties allow their use in various fields, including catalysis, drug delivery, and food applications [1,2,3,4,5]. Various gels based on biopolymers have been prepared using alginates, agarose, starch, gelatin, cellulose, chitosan, and their derivatives, as they have exceptional properties combining efficiency and biocompatibility [6,7]. Especially, mixtures of biodegradable and biocompatible polymers belonging to well-known families of natural polysaccharides (such as cellulose, starch, chitin) have already been approved for use in food industry [8].

Cellulose is the most abundant-in-nature, renewable biopolymer. It has excellent thermal and mechanical properties and biocompatibility [9]; therefore, it has been used in a wide range of applications such as tissue engineering, water purification, encapsulation, and delivery of biocompatible ingredients and as the Supporting Material for immobilizing enzymes. In most cases, cellulose derivatives are used [10,11], because of their physico-chemical properties [12,13,14]. Especially, methylcellulose (MC) and hydroxypropyl-methylcellulose (HPMC) are considered the principal cellulose derivatives [15]. HPMCs are non-ionic cellulose derivatives with methyl (hydrophobic) and hydroxypropyl (hydrophilic) groups added to the anhydro-glucose backbone. HPMCs are water-swelling polymers that provide their promissory usage as vehicles for encapsulation of active ingredients, serving at the same time as humidity absorption agents [16].

The combination of microemulsions and gels [17,18] led to the so-called microemulsion-based organogels (MBGs), which were first reported in 1986 by the groups of Eicke [19] and Luisi [20]. Later, new systems were proposed by our group [21,22], where the combination of a microemulsion and a gel based on HPMC, created very promising MBGs [2]. Several variations of the MBGs have since been prepared, combining HPMC with different ingredients. The microemulsion used can be based on natural surfactants such as lecithin [2,22], the organic solvent can be a vegetable oil or other biocompatible oil [2], and the encapsulated ingredients can be drugs [2], enzymes [23], or other bioactive compounds. Nevertheless, most of the studies were carried out on MBGs formed with an bis-(2-ethylhexyl)sulfosuccinate sodium salt (AOT) in isooctane microemulsion. For the present structural study, we focused on the latter system since, AOT/HPMC is a well-studied system used in several applications [24], and an enzyme was used as a model-encapsulated molecule. Replacement of these ingredients with biocompatible or even edible ones can offer a possibility to further develop interesting applications in various domains.

Therefore, in the present work HPMC-based MBGs were studied in order to clarify their structural properties. Scanning electron microscopy (SEM) was used to examine the morphology of the MBGs and small angle X-ray scattering (SAXS) to detect any residual reverse micelles after microemulsion incorporation in the HPMC matrix and clarify its structure. Moreover, electron paramagnetic resonance (EPR) spectroscopy was applied using hydrophilic, amphiphilic as well as hydrophobic spin probes to investigate the polar and non-polar areas. The enzyme-labelling technique was also followed to investigate the location of the enzyme in the immobilization matrix. The necessity of the presence of an enzyme-carrying microemulsion was tested following an esterification reaction.

Finally, the results were combined to propose a structural model for HPMC MBGs.

## 2. Materials and Methods

Materials: Lipase from *Candida rugosa* Type VII (specific activity of 724 U mg^−1^; 1 U corresponds to the amount of enzyme, which hydrolyzes 1 microequivalent of fatty acid from a triglyceride per hour at pH 7.2 and 37 °C), bis-(2-ethylhexyl)sulfosuccinate sodium salt (AOT), (hydroxypropyl)methyl cellulose (HPMC) (3600–5500 cP), lauric acid as well as the iodoacetamido-TEMPO, and 4-Nitrophenyl butyrate (p-NPB) were obtained from Sigma, Darmstadt, Germany. The spin probe 4-Hydroxy-2,2,6,6-tetramethylpiperidine-1-oxyl (Hydroxy-TEMPO), the spin-labelled doxylated derivatives 5-doxyl stearic acid (5-(1-oxyl-2,2-dimethyl-oxazolidin) stearic acid; 5-DSA), 16-doxyl stearic acid (16-DSA), 12-doxyl methyl stearate (12-DMS), 10-doxyl nonadecane (10-DN), 5-doxyl decane (5-DD), and the spin label 4-(2-iodoacetamido)-2,2,6,6-tetramethyl-1-piperidinyloxy (4-(2-iodoacetamido)-TEMPO) were obtained from Sigma, Darmstadt, Germany. All other reagents were of the highest commercially available purity.

### 2.1. HPMC MBGs Formulation

HPMC gels based on AOT-microemulsions were prepared, as described elsewhere [22]. The weight composition of the studied HPMC-based MBGs ranged in terms of HPMC content, *w_o_* and H_2_O. In a typical experiment, 1 mL of AOT microemulsion (*w_o_* = 15) was prepared by adding the appropriate amount of 0.05 M Tris-HCl buffer or lipase solution to a 0.2 M AOT in isooctane solution. The microemulsion was then added to a mixture of 1 g HPMC and water (1 to 4 g), which was then vigorously stirred with a spatula until homogeneous.

### 2.2. Scanning Electron Microscopy (SEM) Measurements

The morphology of the gels was observed via scanning electron microscopy on a field emission scanning electron microscope (FESEM). The JEOL JSM-7610FPlus Field Emission SEM (Tokyo, Japan) combines two proven technologies—a semi-in-lens detector with integrated electron energy filter (r-filter) and an in-the-lens Schottky field emission gun—to deliver ultrahigh spatial resolution with a wide range of probe currents for all applications (1 pA to more than 200 nA). The JSM-7610FPlus offers true 1,000,000× magnification with 0.8 nm resolution at 15 kV (1.0 nm at 1 kV) and unmatched beam stability, making it possible to observe the fine surface morphology of nanostructures. The JSM-7610FPlus successfully integrates a full set of detectors for secondary electrons, backscattered electrons, energy dispersive X-ray spectroscopy (EDS), WDS, STEM, EBSD, and CL. The samples were freeze-dried before analysis and visualized without any sputtering process.

### 2.3. Electron Paramagnetic Resonance (EPR) Measurements

Electron paramagnetic resonance (EPR) measurements were carried out at ambient temperature, using a Bruker EMX EPR spectrometer (Rheinstetten, Germany) operating at the X-band, and the CW spectra were accumulated using Bruker WinEPR Acquisition Software (Rheinstetten, Germany) for EMX by Bruker Biospin GmbH (Rheinstetten, Germany). Gel samples were placed in an ER 162 TC-Q Tissue cell, Bruker, while aqueous and microemulsion samples were contained in a flat E-248 cell. Typical instrument settings were as follows: center field, 0.3480 T; scan range, 10.0 mT; gain, 5.64 × 10^4^; time constant, 5.12 s; conversion time, 5 ms; modulation amplitude, 0.4 mT; microwave power, 2.147 mW; frequency, 9.8 GHz.

#### 2.3.1. Spin-Probing

In order to obtain a concentration of 10^−3^ M of 5-DSA, 16-DSA, 12-DMS, 10-DN, and 5-DD in the w/o AOT microemulsions, *w_o_* = 15, 1 mL of the microemulsion was added to a tube into which the appropriate amount of amphiphilic or lipophilic probe had previously been deposited. This was obtained by placing 10 µL of a stock probe solution in ethanol (7.8 × 10^−3^ M) in the tube and by further evaporating the ethanol. The same concentration was used for the hydrophilic Hydroxy-TEMPO, which was diluted in water. After gel preparation, the final probe concentration in the systems was 3.5 × 10^−5^ g to 1.4 × 10^−4^ g of spin probe per 1 g of gel. In the microemulsions that were used as a reference system, a concentration of 5 × 10^−4^ M of the probe was used, for comparison reasons.

#### 2.3.2. Spin-Labeling Lipase

The lipase from *C. rugosa* was spin-labelled by the iodoacetamido-TEMPO in a 0.05 M Tris-HCl buffer, pH 7.5 at 25 °C. For this, 45 mg of *C. rugosa* lipase was dissolved in 1.5 mL of 0.05 M Tris-HCl buffer; pH 7.5. Twenty microliters of iodoacetamido-TEMPO 5.3 mM in acetonitrile were added. The reaction mixture was gently agitated for 12 h. The unreacted spin label was removed by extensive dialysis against 0.05 M Tris-HCl buffer, pH 7.5. The spin-labelled enzyme solution was then removed from the dialysis bag and stored in a freezer. A control sample was prepared following the same procedure without the spin-label reagent.

The efficiency of the enzyme spin-labeling was measured by monitoring the loss of lipase catalytic activity towards the hydrolysis of p-Nitrophenylbutyrate (p-NPB). For this purpose, enzyme solution was prepared by adding 30 μL of free or labelled *C. rugosa* lipase in 50 mM Tris/HCl buffer, pH = 8. For the hydrolysis reaction a solution of 1.5 mg p-NPB in 5 mL 2-propanol was added at a ratio 1:9 to the solution of 0.1 g Triton X-100 and 0.025 g Arabic gum containing the enzyme (27 μL). The rate of p-NPB hydrolysis catalyzed by the lipase was followed spectrophotometrically by means of the produced p-nitrophenol (pNP) absorbance at 410 nm. Values reported correspond to the production of pNP versus time. Experiments were performed at room temperature.

#### 2.3.3. Interpretation of the EPR Data

The WinEPR Processing program was used for the processing of EPR experimental spectra. The results reported in this work were analyzed in terms of rotational correlation time, τ*_R_*, order parameter, S, and hyperfine splitting constant, A_N_. The above-mentioned parameters can monitor the dynamics of a spin probe in membranes or in viscous media and the polarity of the microenvironment as sensed by the spin probe molecules [25,26,27,28,29]. The simulations of the EPR spectra were performed using programs in the MATLAB platform (MathWorks), employing the EasySpin toolbox in order to acquire the optimal sample’s τ*_R_*. For the slow-motion regime EPR spectra simulations as well as the fast-motion regime EPR spectra simulations, the “chili” function and the “garlic” function were selected, respectively [30,31]. For the EPR spectra of 10-DND and the spin-labelled enzyme, a two-component analysis computation was used through EasySpin simulations. Simulation of experimental EPR spectra were decomposed into components using SimLabel, a program working on the MATLAB platform, which employs the EasySpin chili function [32]. More details can be found in the Supporting Information section.

### 2.4. Small-Angle X-ray Scattering (SAXS) Measurements

The SAXS experiments were performed on a SAXSpoint 2.0 camera, Anton Paar, Graz, Austria. The equipment is operated at 50 W (50 kV/1 mA) with a Primux 100 microsource, using Cu Kα radiation (λ = 0.1542 nm). The X-ray beam was collimated by scatterless slits. The samples were measured in transmission, and the scattered signal was collected by a 2D Eiger R 1M hybrid photon counting (HPC) detector with 75 µm² pixel size. The investigated *q* range was 0.055–5.2 nm^−1^. *q* is defined by *q* = 4π (sin θ)/λ, with 2θ being the scattering angle with respect to the incident beam and λ the wavelength of the X-rays. The exposure time for each sample was 10 min. Microemulsions were measured in a 1 mm diameter quartz capillary, and MBG samples were measured in a multiple paste holder (Kapton windows). All measurements were performed at 25 °C. The measured scattering curves were corrected for transmission losses and put on absolute scale.

## 3. Results

HPMC MBGs have been successfully used as biocatalysts [24] or as drug carriers [2]. In both cases, it has been observed that the nature and amount of each ingredient influences the catalytic activity and the release profile of the encapsulated compound, respectively. A characteristic example has been reported [24], where the different water concentrations affect the efficiency of the system by means of catalytic activity. Those alterations of the gel’s composition are able to cause changes to the structure, thus creating the urge to understand the morphology of the HPMC MBGs. The structural study can offer an insight on how each component affects the final matrix and therefore the behavior and properties of the system. In addition, an extensive structural characterization will contribute to the rational development of various systems according to the application.

### 3.1. Catalytic Activity

**Figure 1:**
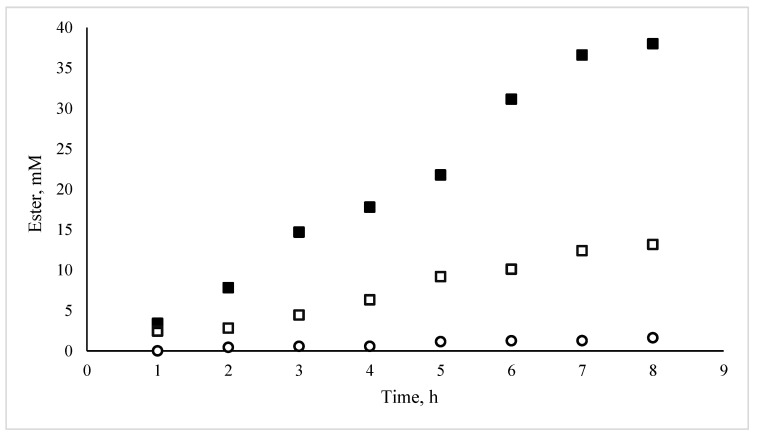
Effect of microemulsion-based gels (MBG) preparation on the rate of propyl laurate synthesis, catalyzed by *C. rugosa* lipase. [1-propanol], [lauric acid]: 100 mM, each; isooctane as solvent (10 mL); *C. rugosa* content per MBG 0.3 mg. (■): (hydroxypropyl)methyl cellulose (HPMC)-based MGB; (□): HPMC matrix with the enzyme and the microemulsion added separately; (○): HPMC matrix with the enzyme (no microemulsion).

Previous works have demonstrated that the HPMC-based MBGs hosting lipases are excellent biocatalysts [33,34,35]. In order to investigate the contribution of the microemulsion in the final catalyst, three different systems were prepared using 1 g HPMC, 2 g H_2_O with or without 1 mL of AOT microemulsion (0.2 M, *w_o_* = 7.5). For the first system, the enzyme was added in the microemulsion, which was then mixed with the HPMC/water mixture. In the second one, the enzyme was added in the water used to dissolve HPMC prior to the addition of the microemulsion, whereas in the third one, there was no microemulsion in the gel. The total amount of the enzyme in each system was 0.3 mg. The experimental protocol was designed aiming to observe the differences of the encapsulated and “free” lipase inside the matrix. The results are shown in Figure 1. It can be noted that in the absence of a microemulsion, the enzyme is practically inactive. Then, the activity is much more important when the enzyme is included in the microemulsion when added to the MBG than when it is added separately. Obviously, the enzyme is in need of the microemulsion ingredients. This can be attributed to the surfactants that create protected surfaces between the organic and water domains. Furthermore, the necessity of the microemulsion as a carrier of the enzyme in the final system is essential, as the enzyme does not come in contact with the organic phase, which would lead to the protein denaturation.

### 3.2. Morphological Analysis

**Figure 2:**
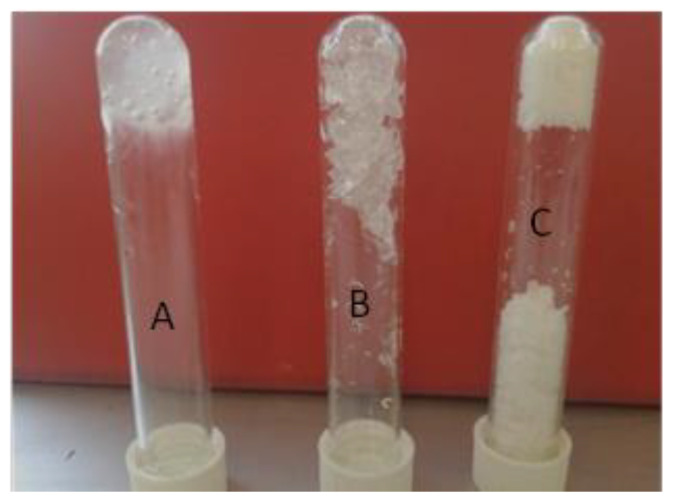
HPMC-based MBGs of different water content. System A: HPMC = 18% *w*/*w*, H_2_O = 71% *w*/*w*, µE = 11% *w*/*w*; System B: HPMC = 28% *w*/*w*, H_2_O = 55% *w*/*w*, µE = 17% *w*/*w*; System C: HPMC = 44% *w*/*w*, H_2_O = 43% *w*/*w*, µE = 13% *w*/*w*. µE: AOT microemulsion *w_o_* = 15. Tubes are reversed to visualize their sticky shape.

Three HPMC-based MBGs were studied with different weight compositions (Figure 2), namely, System A containing 71% *w*/*w* water, System B containing 55% *w*/*w* water, and System C containing 43% *w*/*w* water. The exact compositions of the systems are shown in Table 1. Since HPMC with microemulsion forms a gel matrix within a narrow window of polymer mass fraction [22], the systems studied here have been chosen to represent the whole range of polymer/water ratios that can lead to final gel matrix formation. It should be mentioned here that the chosen systems have been used in previous studies that determined the state of water that they contain [36].

**Figure 3:**
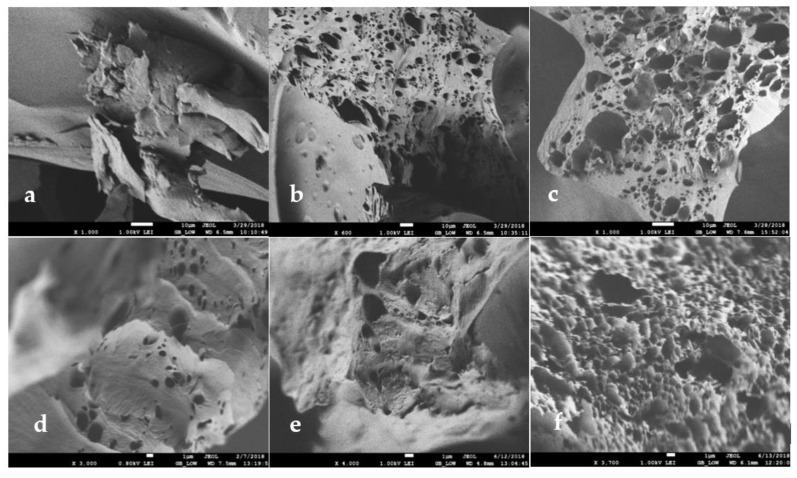
SEM images of freeze-dried HPMC-based MBGs. (**a**) System A prepared without organic components; (**b**) System A prepared with isooctane; (**c**) System A prepared with 0.1 M AOT µE; (**d**) System B prepared with 0.1 M AOT µE; (**e**) System C prepared with 0.1 M AOT µE; (**f**) System B prepared with 0.2 M AOT µE. µE = microemulsion.

The morphology of the different systems was observed via SEM images revealing the formation of a three-dimensional network. In order to monitor the role of each component of the microemulsion on the gel structure, images were taken for HPMC/water mixture without any organic solvent. Then pure isooctane was added to a HPMC/water mixture corresponding to System A, and finally, AOT microemulsion was used as the organic component to form System A. A comparison of the images taken for the freeze-dried systems reveals that when no organic solvent is used the matrix appears to be compact, without a network of pores (Figure 3a). When isooctane is added, the appearance of pores in the coherent, otherwise, material can be observed (Figure 3b). This could be attributed to the fact that the organic solvent congregates in the surrounding polar environment assembling enclaves that after freeze-drying leave the observed pores. The addition of micelles in the organic solvent by using microemulsion instead of pure isooctane, leads to a more porous matrix, although the water content does not change (Figure 3c). The effect of water content was also studied. For the three systems studied, namely Systems A, B, and C, the images are shown in Figure 3c–e, respectively. As can be seen, the addition of water facilitates the appearance of pores. Increasing water, the pores population increases (Figure 3e to 3c, respectively) until a sponge-like structure can be seen for the system with 71% *w*/*w* water (System A, Figure 3c). We can also notice a broadening of the pores for the system with higher water content. Similar results for the structural investigation of gelatin MBGs were also observed by Dandavate and Madamwar. Gelatin MBGs showed pore widening after use, which was attributed to the accumulation of water molecules that cause swelling of the assumed coexisting w/o microemulsion droplets [37].

Moreover, the influence of the surfactant concentration was studied for System B based on microemulsion with 0.1 M or 0.2 M AOT concentration (Figure 3d,f). As can be seen, when the surfactant concentration is higher the gel appears to have a more uniform network consisting of more, smaller, and evenly distributed pores. The same effect of smooth and uniform network formation was observed over the addition of polyethylene glycol (PEG) on gelatin MBGs [38].

### 3.3. Interfacial Properties

In order to study the properties of the interfaces as well as the polar or non-polar areas of the constructed gels, Electron paramagnetic resonance (EPR) spectroscopy was engaged using different probes, polar, non-polar, and amphiphilic ones.

#### 3.3.1. Hydrophilic Spin-Probe

The hydrophilic probe Hydroxy-TEMPO was used to study the properties of the polar areas of the MBGs. For this purpose, the probe was dissolved (a) in the water pools of the microemulsion, which was then added to the HPMC/water mixture or (b) in the water used to hydrate HPMC prior to the addition of the microemulsion. The following reference systems were chosen: (i) microemulsions formed with water and 0.1 M or 0.2 M AOT, with *w_o_* = 15 or *w_o_* = 7.5, respectively, (ii) HPMC/water mixtures (ratios corresponding to Systems A, B, and C), and (iii) HPMC with water and isooctane (without AOT). The results are presented in Table 2. As Table 2 shows, the *A_N_* parameter value obtained in microemulsion ((AOΤ) = 0.1 Μ, *w_o_* = 15) reflects a less polar microenvironment comparing the one obtained in water. A lower hyperfine splitting constant value also occurs when a different microemulsion is used ((AOΤ) = 0.2 Μ, *w_o_* = 7.5). This can be attributed to the different nature of the water molecules that form the microemulsion’s water pools, comparing to the ones in bulk water [39,40]. On the other hand, τ*_R_* values calculated for the spin-probe Hydroxy-TEMPO molecules in both microemulsions used showed lower mobility, since they are higher than the value obtained in aqueous solution. This reflects the existence of bound water in the microemulsion’s water core.

A remarkable increase in immobilization of the hydrophilic spin probe occurs when incorporated into the gel via solubilization in the microemulsion. Immobilization, as expressed by *τ_R_* values, increased when the water content of the gel decreased from 71% to 55% and to 43% *w*/*w*, respectively (Systems A, B and C), while at the same time, the polarity as expressed by hyperfine splitting constant decreased. Quite similar *τ_R_* and *A_N_* values occurred for System C independently on the microemulsion used (Table 2, Systems C and C^†^).

Table 2 also shows the corresponding *τ_R_* and *A_N_* values for the same spin-probe incorporated in the gels prior the addition of the microemulsion. Comparing the obtained values, it becomes obvious that the microenvironment polarity as “sensed” by the hydrophilic spin probe as well as its mobility are quite similar in both cases, when the spin probe molecules are either incorporated in the water pool of the AOT microemulsion used for the construction of the gel or incorporated directly in the “outer” water. The polarity slightly decreased when the water content of the gel decreased, and the calculated values are much higher than the ones calculated in the microemulsion. The values obtained for gels with the same water content can be considered equal within experimental error, regardless how the spin-probe was incorporated in them. In addition, both *τ_R_* and *A_N_* values of this hydrophilic spin probe indicate a linear dependence on the amount of water as can be seen in Supporting Information Figures S1 and S2. Furthermore, the values of the *τ_R_* and *A_N_* for the same spin-probe in the HPMC/water or HPMC/water/isooctane mixtures (Systems A*, B*, C*, and C**) follow a similar pattern showing similar behavior.

This finding gives strong evidence that no form of microemulsion droplets exist after the addition of the microemulsion in the HPMC/water mixture, and consequently, the micellar water is mixed with the “outer” water and absorbed by the HPMC network.

#### 3.3.2. Amphiphilic Spin-Probes

Table 3 presents the *τ_R_*, *S*, and *A_N_* values obtained when the amphiphilic spin-probe molecules 5-DSA and 16-DSA were incorporated in the three HPMC-based MBGs examined. These spin-probes were incorporated in the gels via the AOT microemulsion, where they were previously solubilized. The calculated values shown in Table 3 for both probes correspond to the expected locations of n-DSA molecules located in the interface of w/o microemulsions, where n refers to the carbon atom of the fatty acid to which the doxy1 group is anchored. Indeed, for 5-DSA molecule where the paramagnetic group is closer to the carboxyl group, i.e., deeper in the water/oil interface, a stronger mobility restriction is evident giving higher *τ_R_* and *S* values, while being closer to the water core leads to higher *A_N_* values [3,41].

**Figure 4:**
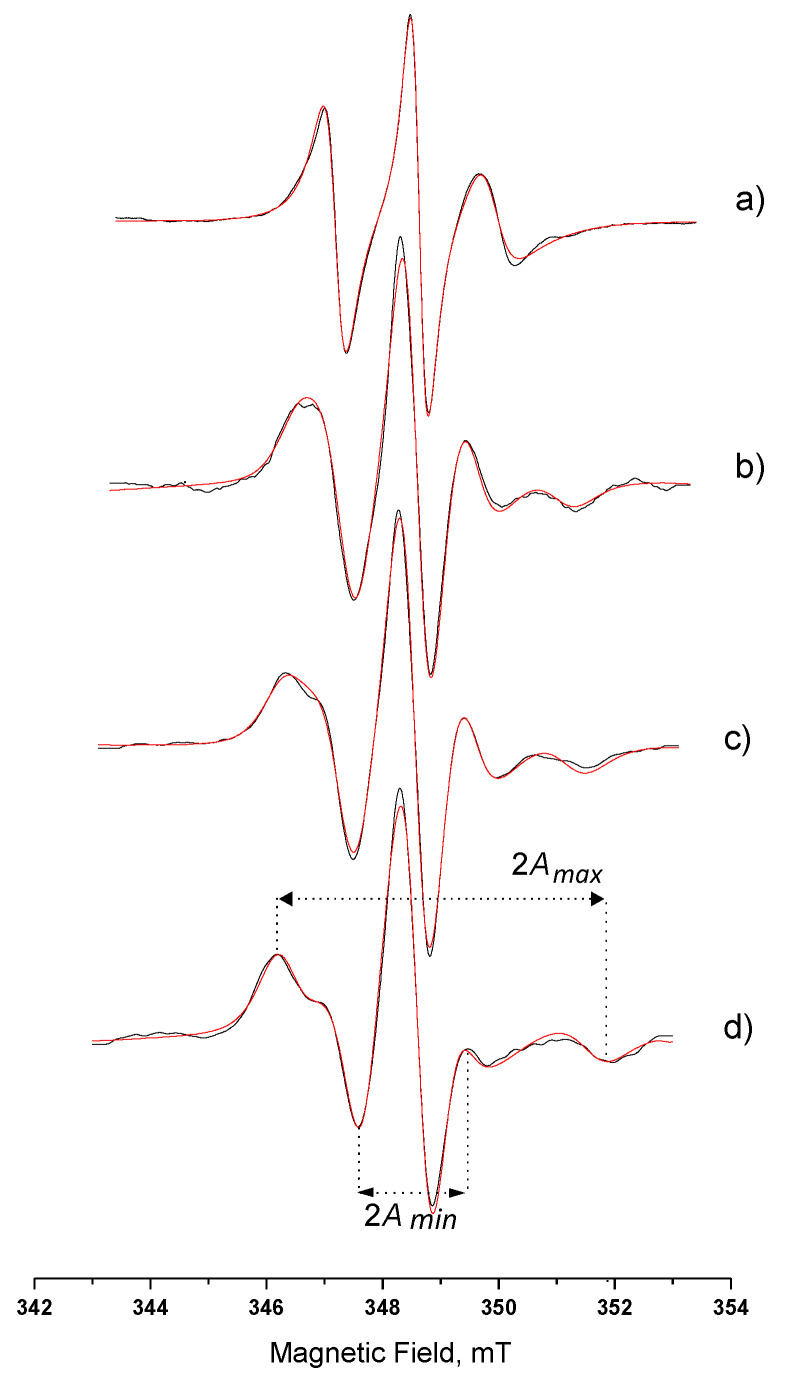
Spectra of 5-DSA in (**a**) AOT microemulsion *w_o_* = 15; (**b**) System A; (**c**) System B; (**d**) System C. Βlack line, experimental; red line, simulation. (A_max_ and A_min_ as described in the Supporting Information section).

Figure 4 shows the experimental and the simulation spectra of 5-DSA in AOT microemulsion and in Systems A, B, and C. More pronounced immobilization (*τ_R_*) occurred when amphiphilic spin probes were involved. As can be seen in Figure 4, the alterations of spectrum characteristics are obvious. The observed differences between spectra obtained in microemulsion and in MBGs can be attributed to the immobilization of the spin probe in the AOT layer in the gel matrix. As the gel water content decreased, Figure 4b–d, the outer hyperfine splitting, 2A_max_, increased, while the 2A_min_ decreased. *τ_R_* values appear to have a drastic increase when the microemulsion is incorporated in the HPMC/water matrix (Table 3) corresponding to the slow-motion regime. The order parameter value, S, is also increased, due to the highly ordered arrangement of the amphiphilic probe molecules among the surfactant molecules. This gradually increasing high-ordered state occurs both at that depth of the interface corresponding to the 5th carbon atom (5-DSA) and to the 16th carbon atom (16-DSA), as it can be seen in Table 3. It is also clear that the *τ_R_* and *S* calculated values for 16-DSA in the final gels are much lower than the corresponding values for 5-DSA; therefore, 16-DSA molecules are less restricted. This could be explained by the closer location of the paramagnetic ring of 16-DSA to the oily phase than the carbon chains of AOT and 5-DSA. The calculated values for both probes follow the same pattern; however, for 16-DSA, they correspond to the fast motion regime. Spectra of 16-DSA can be seen in Supporting Information Figure S3.

The calculated polarity of the microenvironment of the paramagnetic ring of 5-DSA and 16-DSA as expressed by *A_N_* values (Table 3) follows a different pattern, as the water decreases in the MBGs. More specifically, the hyperfine splitting constant issued from the 5-DSA spectra increases, while the one corresponding to 16-DSA decreases. Plots of the above mobility (*τ_R_*) and hyperfine splitting constant (*A_N_*) data of the amphiphilic probes versus the water content appear to follow linearity for the 5-DSA, while the ones for 16-DSA deviate remarkable from linearity. The behavior of 16-DSA is similar to the one of the lipophilic probes, the alkane 5-DD (Supporting Information Figures S1, S2, and S4).

Given the strong binding of the polar head –COOH of both DSA’s with the water molecules, in the case of 5-DSA, the paramagnetic ring is closer to water molecules, and the results indicate that it “senses” an increased polarity, showing a smaller distance between the hydrophobic tails of AOT. As water reduces progressively from Systems A to C, the water/isooctane interface seems more rigid, giving remarkably less freedom in the probe’s mobility and the two amphiphilic probes tend to detect quite different polarity regarding the position of the paramagnetic ring on their aliphatic chain. This can be explained if we assume a tight, almost parallel arrangement of the surfactant molecules, which is in agreement with the assumption of channel formation of AOT layers.

#### 3.3.3. Lipophilic Spin-Probes

**Figure 5:**
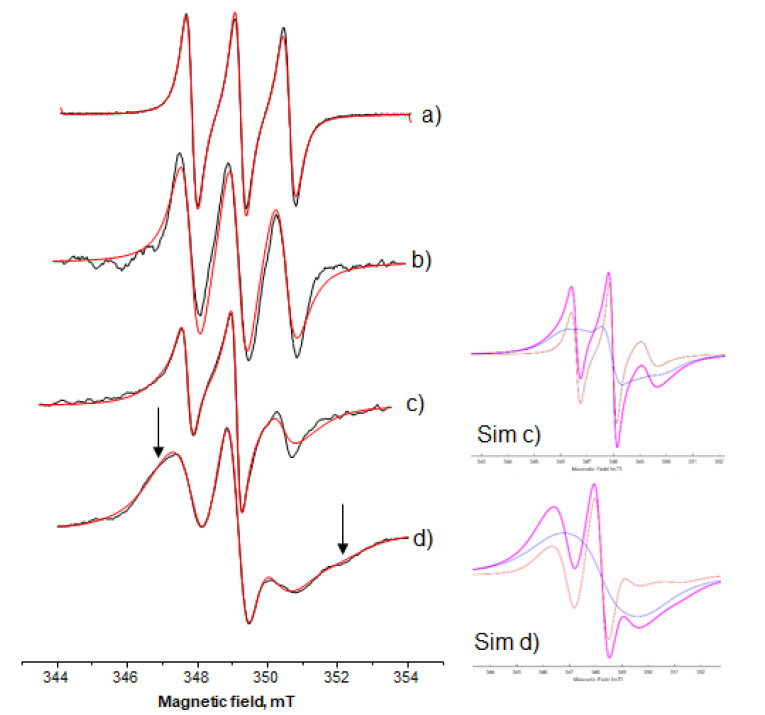
Ten-doxyl nonadecane (10-DND) in (**a**) AOT microemulsion *w_o_* = 15; (**b**) System A; (**c**) System B; (**d**) System C. Black line, experimental; red line, simulation. In the inserts show the simulations (violet line) of the spectra (**c**) and (**d**) (Sim (c) and Sim (d), respectively) and the analysis of its components (mobile: orange line, immobile: blue line).

In order to examine the role of the organic phase, i.e., isooctane introduced through the AOT-isooctane microemulsion into the HPMC-based MBGs, three lipophilic spin probes were followed, namely, 10-doxyl nonadecane (10-DND), 5-doxyl decane (5-DD), and 12-doxyl methyl stearate (12-DMS). Considering the two spin-probes, 10-DND and 12-DMS, a quite similar behavior was detected. Figure 5 shows the experimental as well as the relative simulation spectra of 10-DND in AOT microemulsion and in Systems A, B, and C. From these spectra, it can be observed that as the water decreases the spectral characteristics show two main spectral shape alterations. Firstly, the spectrum of 10-DND recorded in the AOT microemulsion is a typical spectrum of nitroxide, showing three equal peaks (first derivate of the original spectrum), which is characteristic of fast molecular motion in a non-polar medium, in the EPR spectroscopy time scale. When 10-DND was embedded in the final gel structure, the three peaks became progressively (i) unequal and (ii) in different position as regards the base line, i.e., a scale shape. The arrows in Figure 5 show spectral characteristics that indicate immobilization as the water decreases (broadening in the low field—splitting in the high field), and this is more pronounced when the water content is low (System C). For the 10-DND spectra analysis, we applied computer-aided (MATLAB/EasySpin/SimLabel) single component simulation. When this probe was embedded in the AOT microemulsion and System A, the calculated rotational correlation time, *τ_R_*, values were 0.06 ns and 0.88 ns, respectively (Figure 5a,b). However, when the probe was embedded in Systems B and C a two-component analysis gave more consistent results during the fitting process. The rotational correlation time, *τ_R_*, calculated values for System B were 1.45 ns and 5.86 ns for the mobile (22%) and the immobile component (78%) (Figure 5c). The *τ_R_* calculated values for System C were 3.59 ns and 6.43 ns for the mobile (21%) and the immobile component (79%), respectively (Figure 5d). Simulation trials using two-component analysis for System A gave a 92% mobile component with *τ_R_*, value 0.08 ns and an 8% immobile component with *τ_R_*, value 8.43 ns. These results give evidence of a dramatic alteration of the structure of the final gel systems between System A on one hand and B, C on the other hand. The calculated hyperfine splitting constant values, *A_N_*, as “sensed” by the 10-DND probe were 14.14 ± 0.04 × 10^−4^, 13.9 ± 0.03 × 10^−4^, 14.01 ± 0.02 × 10^−4^, and 14.12 ± 0.03 × 10^−4^ for the AOT microemulsion and the three systems A, B, and C, respectively.

The second characteristic is indicative that, as the water decreases, the spin system undergoes an increasing spin–spin interaction [42]. This is a result of high local spin probe concentration or partially molecular aggregation of the spin probes. This is confirmed by the simulation analysis of the spectra shown in Figure 5c,d. The immobile component is also reflecting the accumulated 10-DND molecules (Figure 5, the blue line of the decomposed spectra).

The spectra of the second hydrophobic spin probe, namely, 5-doxyl decane (5-DD) recorded in the same systems, follow a different pattern. This smaller molecule also shows gradual immobilization in the fast motion region but did not show high local concentration. More specifically, the rotational correlation time, *τ_R_*, values calculated were 0.03 ns, 0.62 ns, 1.26 ns, 2.52 ns; the order parameter, S, values were 0.04, 0.06, 0.08, 0.17; and the measure of hyperfine splitting constant, *A_N_*, values were 14.36 × 10^−4^ T, 14.67 × 10^−4^ T, 14.5 × 10^−4^ T, 14.05 × 10^−4^ T in the AOT microemulsion and in Systems A, B, and C, respectively.

This deferent behavior of the two lipophilic probes is interesting and can be explained by the assumption that a remarkable part of the organic solvent, i.e., the isooctane molecules, is absorbed through the HPMC-based MBG lattice in the hydrophobic regions of propyl- and methyl-groups of the HPMC molecules. This absorption is most effective when water content is low (Systems B and C). A similar result was reported for gelatin–AOT organogels [43]. The larger molecules of 10-DND and 12-DMS probably cannot diffuse through the HPMC/water matrix and therefore are accumulated inside the channels of the remaining organic solvent, while the smaller molecule of 5-DD preferably follows the behavior of isooctane. The relative indicative spectra for the 5-DD and the 12-DMS are presented in the Supporting Information section, Figures S3 and S4.

#### 3.3.4. Spin-Labelled Lipase

While these gels have been used as immobilization matrices for several lipases, among which is the lipase VII from *Candida rugosa*, their structure and the location of the enzyme is yet to be described. In order to investigate the possible conformational changes in the lipase and to clarify its location when immobilized in the gels, the spin-labelling technique was applied and followed by EPR spectroscopy using the iodoacetamido-TEMPO as a spin label. The iodoacetamide group has an iodine leaving group and attaches rather selectively to thiol groups, -SH, which are present in cysteine residues, forming a stable irreversible thioether bond. Spin labelling of other amino acids cannot be excluded if the reagent is in excess and in acidic pH. The chosen lipase has five cysteine residues at the positions 60, 97, 217, 268, and 277. Four of them are linked as pairs by a disulfide bridge, Cys60-Cys97 and Cys268-Cys277, while Cys217 is a single cysteine residue with the free -SH group, not bound with an S-S bridge [44,45,46]. Enzyme labelling was verified following the hydrolysis of p-NPB. The labelled enzyme lost 80% of its activity, while the non-labelled enzyme that followed the same treatment lost only 20% of activity.

**Figure 6:**
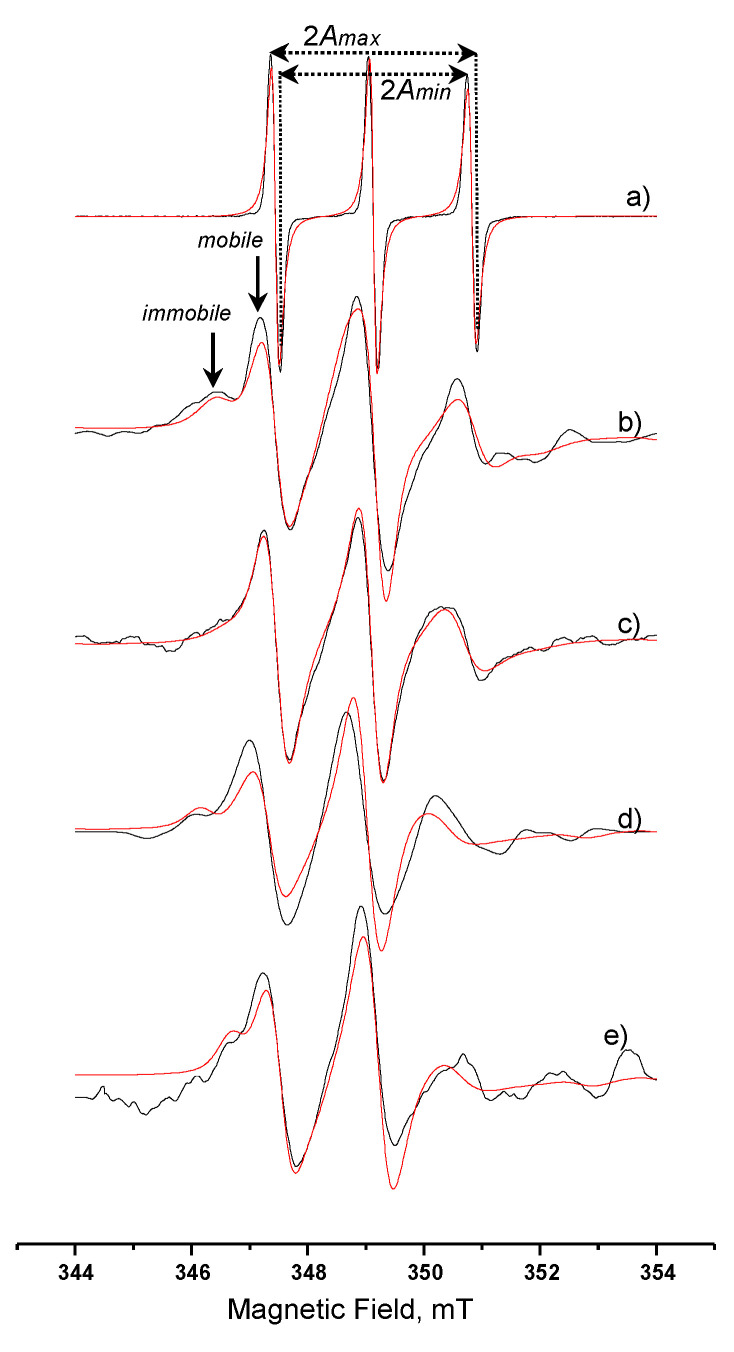
EPR spectra of (**a**) free spin-label iodoacetamido-TEMPO in aqueous solution; and spin-labelled *C. rugosa* lipase in: (**b**) aqueous solution; (**c**) AOT microemulsion *w_o_* = 15; (**d**) System A; (**e**) System B. Black line, experimental; red line, simulation.

Figure 6 shows the spectra of (a) free spin-label recorded in aqueous solution and spin-labelled lipase recorded in (b) aqueous solution, (c) AOT microemulsion *w_o_* = 15, (d) System A, (e) System B. The respective calculated relative *τ_R_*, S, and A_N_ values are presented in Table 4. The spectrum characteristics of the free spin label in water (Figure 6a), showing three narrow peaks of equal height, is indicative of highly fast movement. The spectrum of the labelled enzyme in aqueous solution, Figure 6b, shows an “immobilized” part and a “mobile” one (indicated with arrows). It is assumed that the spin label can be traced in two different states, one strongly bound to the cysteine residue and another one with a weaker bond on the surface of the protein, sensing thus, a different microenvironment [47]. This observation leads us to use a two-component computation analysis. It was calculated that in aqueous solution the immobile component was 44%, while the mobile one was 56%. The calculated *S* value (0.41) is an overall estimated value for the ordering behavior of the bound on the protein molecule spin label. The calculated *A_N_* value shows an environment less polar than water, however, the polarity is still quite high.

The spectrum in Figure 6c (spin-labeled lipase in microemulsion) is indicative of a more relaxed form of the enzyme. The peaks at 346 and 353 mT that correspond to the strongly bound spin label almost disappeared. More specifically, the immobile and mobile component ratio is altered to 20% and 80%, respectively, followed by altered rotational correlation time values (Table 4). The increase in the ratio of the relaxed form of the enzyme can be explained considering that the environment in the microemulsion is less polar due to the presence of AOT molecules, and this is in agreement with the calculated *A_N_* value. It should be noted here that the calculated percentages do not correspond to the actual weight percentages of the two states of the spin label, but they can be considered as the average movement of the spin-label/lipase complex in the AOT microemulsion’s water-pool. The *S* value calculated from the spectral characteristics is also lower than the one in aqueous solution, and this confirms the above observations.

When the spin-labelled lipase is incorporated in System A, the spectral characteristics are again indicative of two different states of spin label. From Table 4, we can see that the A_N_ value lays between the values obtained in aqueous solution and in microemulsion. The values of the rotational correlation time for the immobile and mobile component indicate that a quite strict environment affects the movement of the lipase. The immobilization is also confirmed from the order parameter value S. The spectrum for the spin-labelled lipase in System B (Figure 6e) is similar to the one recorded in System A (Figure 6d). The relative calculated values, as they are reported in Table 4, indicate a higher immobilization as both *τ_R_* values increase with a percentage of 43% and 57% for the two states of the spin label, respectively. The hyperfine splitting constant value, *A_N_*, also lays between the values obtained in aqueous solution and in the microemulsion. It is obvious that the simulation spectra in Figure 6d,e are less well fitted to the relative experimental spectra. Considering the multi-compartmental structure and the variable stiffness of MBGs under investigation, we can say that there are many immobilized states of the lipase molecule and this fact implies more than two states of the anchored spin label. This also consequently results in the increase in the Gaussian participation in the simulation spectra.

Taking into account this study, we can assume that there is evidence that the lipase molecules in both gels tested are preferably located close to the AOT interface. In parallel, less water content in the gel leads to more restricted lipase molecules.

### 3.4. Small-Angle X-ray Scattering (SAXS) Measurements

**Figure 7:**
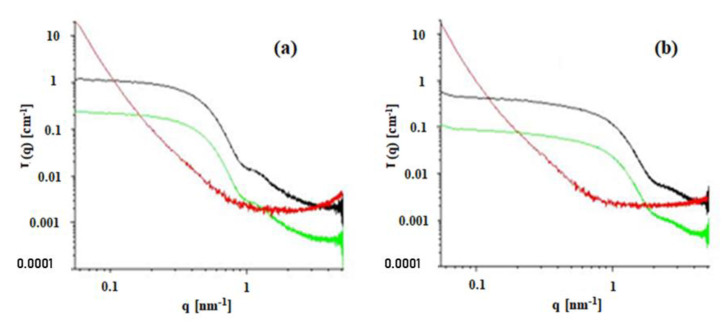
Scattering curves on absolute scale for HPMC-based MBGs (System C, red curves) and microemulsions with 0.05 M (**a**) and 0.2 M (**b**) AOT (black curves). A factor of 0.2 was applied to the microemulsions (green curves) to show the expected contribution of the micellar signal in the gel.

SAXS was used to verify whether there are residual reverse micelles after incorporation in the MBGs as well as to clarify our perspective of the MBGs morphology. Data shown in Figure 7 were transmission-corrected and put on absolute scale [48]. The Kapton background was subtracted from the curves of the gel samples. To approach the amount of microemulsion in the MBG, the intensities of the microemulsions have been scaled to 20% scattering intensity (green curves). The microemulsions show clear scattering features of nanometer-sized droplets. These can be analyzed in detail by the indirect Fourier transformation [49]. The microemulsion containing 0.05 M AOT forms spherical micelles of approximately 11 nm in diameter, while the one containing 0.2 M AOT has a mean diameter of approximately 5 nm. This is the reason for the decay at lower q-values for the 0.05 M AOT sample. The comparison of the microemulsions with the corresponding gel samples shows no remaining contribution of the microemulsion signal, confirming, thus, EPR findings.

Of the available models that were tested against SAXS data, the so-called Gel Fit Model (in SasView) was used to gain further knowledge of the gel properties, since this was the model that best fitted our data. Therefore, the data were fit to the Correlation Length Model shown in Equation (1):

I(Q) = A/Q^n^ + C/[1 + (Qξ)^m^] + B (1)

The first term describes the Porod scattering from clusters, and the second term is a Lorentzian function describing scattering from polymer chains. The two multiplicative factors A and C, the constant background B, and the two exponents n and m are used as fitting parameters. The final parameter ξ is a correlation length for the polymer chains [50,51], while the Porod and Lorentzian exponents are used for the fractal structure and polymer/solvent interaction, respectively.

The structural parameters obtained from the Correlation Length Model (Table 5) for the MBGs formed with 0.05 M and 0.2 M AOT microemulsion show that the correlation length (ξ) increases with the increased surfactant concentration. Higher AOT concentration in the microemulsion—and as a result, in the final gel—leads to an increased entanglement length of HPMC, creating an environment of higher stiffness in comparison to the systems prepared with isooctane instead of a microemulsion. The equation’s first term (A/Qn) describes the Porod scattering from clusters where the network collapses and forms compact particle-like structures. The Porod exponent (n) values (Table 5) calculated for the HPMC/water mixture in the presence or absence of isooctane are similar. The n value increases when AOT is added via the microemulsion with further increase for higher AOT concentration, indicating increased compactness of the formulated clusters. In contrast, the Lorentz exponent (m) does not change (Table 5), showing that while the compactness of the cluster grows, that of the network remains unchanged. This indicates a stronger effect of AOT on the local nanostructure. The increased compactness of HPMC clusters in MBGs prepared with 0.2 M AOT microemulsion may create an environment, where the organic solvent can be more easily distributed in comparison to the MBGs prepared with 0.05 M AOT, due to the space that the compact clusters leave among them. As a result, the evaporation of the solute prior SEM observations creates the pores, which as mentioned before, are more uniform (Figure 3f).

## 4. Discussion

The use of HPMC network combined with a microemulsion as a successful enzyme immobilization matrix has led to the investigation of the system’s structural characteristics. The fact that those gels have shown a catalytic activity only in the presence of microemulsion, with much better results when the enzyme is introduced to the gel entrapped in the microemulsion rather than separately, has given the motive to study the structure of such systems in order to gain basic knowledge regarding the use of the biocatalyst. The preparation of such gels differs from the gelatin-based gels studied in the past [19,20,52,53], where the polymer is dissolved in the water of the microemulsion rather than the preparation of a gel that would absorb the microemulsion [35]. Luisi’s group [20] prepared MBGs by adding solid gelatin to an already prepared AOT microemulsion. On the other hand, the groups of Eicke [19] and Robinson [52] prepared MBGs by adding an aqueous gelatin solution to an AOT/oil solution. On the contrary, HMPC-based MBGs studied here are prepared by adding AOT microemulsion to an HPMC/water mixture [21,22]. Nevertheless, due to the similarity of application and the use of polymers and microemulsions for the formulation, questions arose about whether the models proposed in the past could also apply to HMPC-based gels.

According to our EPR and SAXS findings, after incorporation in the gel, the microemulsion cannot be detected in the form of reverse micelles. There is no distinction between the aqueous phase of the microemulsion and the water used to hydrate HPMC, therefore, independently of how the water is introduced in the gel matrix, it is rearranged. The use of lipophilic spin probes to obtain EPR spectra leads to the conclusion that the organic solvent seems to congregate. This is obvious not only by the higher immobilization detected, but mainly by the increased local concentration that the longer carbon chain probe molecules exhibit. Moreover, according to the SEM images recorded for HPMC gels in the presence and absence of organic solvent, it becomes obvious that the organic solvent is essential for the formation of pores or channels.

The results obtained by using amphiphilic probes and EPR spectroscopy lead to the conclusion that in the MBGs, the molecules of the surfactant are still ordered. However, their arrangement is different than the one they present in the initial microemulsion, since there are indications that the AOT tails are packed more tightly showing a parallel arrangement. The surfactant molecules create a layer via which the enzyme can be protected in the aqueous phase of the system, while a non-polar channel on the other side of the layer ensures the diffusion of the substrates. Therefore, the location of the enzyme near the surfactant molecules allows the protein to always be close to the reaction’s substrate. On the other hand, when the enzyme is introduced in the MBG via the HPMC/water mixture before adding the microemulsion, although the surfactant-coated channels are still formed, the enzyme might be located anywhere in the matrix and not necessarily close to the channels. Therefore, although it is still active, the observed activity is significantly lower. These findings clarify the necessity of the enzyme-containing microemulsion for the construction of the biocatalyst, even though after its incorporation the initial structure of the microemulsion droplets disappear.

Combining our previous work [36]—in which following Differential Scanning Calorimetry (DSC), different types of water were identified in the HPMC MBGs—with the present findings, we can conclude that the identified type of water with the strongest interactions can be water molecules bound strongly on the heads of the surfactant molecules. Nevertheless, it has been proven before [54] that two molecules of water are more tightly bound to the heads of AOT than the rest of the water of hydration. The identified interfacial water [36] can be the rest of the AOT hydration molecules that accumulate on the side of the heads of AOT molecules that cover the channels formed by the organic solvent. As the water content in the system increases, free, bulk water appears where the water molecules can be dissolved among the polymer chains presenting very weak interactions with the matrix. This is in agreement with the findings of SEM study, where the system with a higher amount of water leaves broader pores after solute evaporation. Moreover, EPR study of lipophilic probes allows the assumption that the higher the water content of the MBG (with bulk-like water appearance) the lower the organic solvent that dissolves in the HPMC mesh.

Finally, in the present study, SEM images and SAXS profile prove that the surfactant concentration is crucial for the distribution of the organic solvent in the gel matrix.

**Figure 8:**
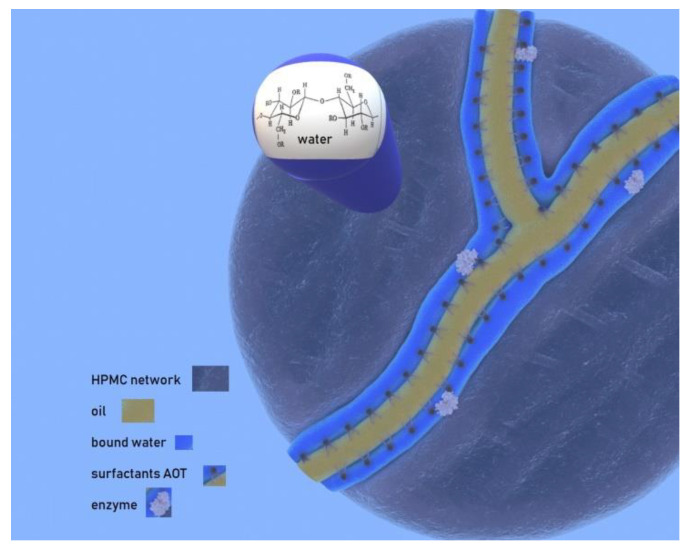
Proposed structural model for HPMC-based MBGs.

Taking all these into account, we propose a structural model presented in Figure 8 (corresponding to System B), according to which the organic solvent forms channels (Figure 8, yellow area) surrounded by surfactant molecules. These surfactant-coated channels are surrounded by water molecules among which the enzyme could be located (Figure 8, blue area). This water layer is narrower for MBGs with low water content and wider for MBGs with higher water content. The water can be found bound on the heads of the surfactant molecules or bound on the substitutes of the cellulose molecules. In systems with higher water content, a lower amount of organic solvent and higher water amount dissolves in the HPMC mesh, where water accumulates as bulk-like water. The opposite takes place in low water systems.

Overall, the results of the present work led to understanding the structure of such systems that have been so effectively used as biocatalysts. The applied techniques offer an overall view of the matrix created from the polymer, the surfactants of the microemulsion, the polar and non-polar solvents of the system as well as the enzyme.

## 5. Conclusions

Although the HPMC MBG has been successfully used as matrices for enzyme immobilization or biocompatible ingredients encapsulation, little is known about the structure of these systems and the role of their components. Deeper knowledge would aid the catalyst optimization as well as ease the encapsulation of bioactive compounds. Therefore, in the present study, we applied SEM to study the morphology, SAXS to detect the presence of droplets, and EPR to investigate the polar and non-polar areas of the matrix. Furthermore, we spin-labelled a model enzyme to investigate the possible conformational changes i the lipase and to clarify its location when immobilized in the MBGs. It should be noted here that this is the first work to combine these techniques to demonstrate an insight of the structure of these immobilization matrices. According to our findings, an organic solvent-based microemulsion is essential to form the MBGs; however, after the preparation procedure, the microemulsion droplets can no longer be detected. Our study leads to a proposed structural model for HPMC-based MBGs, according to which in the HPMC network the organic solvent forms channels that are surrounded by surfactant molecules. These surfactant-coated channels are covered by water molecules among which the enzyme can be located. No microemulsion droplets can be detected after its incorporation in the MBG. The channels could facilitate the diffusion of the substrates during a reaction. Taking into account our findings on how water, the organic solvent, and the surfactant influence the structure of the catalyst, in the future it will be easier to optimize the system according to the application that it will facilitate.

## Figures and Tables

**Table 1:** Weight composition of the studied HPMC-based MBGs.

MBG	HPMC	H_2_O	µE
	% *w*/*w*		
System A	18	71	11
System B	28	55	17
System C	44	43	13

µE = AOT/isooctane microemulsion.

**Table 2:** *τ_R_* and *A_N_* values of hydrophilic spin-probe Hydroxy-TEMPO in water, AOT microemulsions, and HPMC-based MBGs systems.

Hydrophilic Probe Hydroxy-TEMPO		
System	*τ_R_*, ns	*A_N_*, 10^−4^ T
Water	0.03 ± 0.00	17.49 ± 0.04
System A*	0.07 ± 0.01	17.29 ± 0.11
System B*	0.15 ± 0.01	17.25 ± 0.03
System C*	0.40 ± 0.01	17.07 ± 0.04
System C**	0.40 ± 0.06	17.07 ± 0.03
(AOT) = 0.1 Μ µE *w_o_* = 15	0.13 ± 0.01	16.25 ± 0.02
(AOT) = 0.2 Μ, µE *w_o_* = 7.5	0.21 ± 0.01	15.84 ± 0.01
*Probe incorporated in the HPMC-based MBGs* via *the AOT microemulsion*		
System A	0.06 ± 0.01	17.31 ± 0.01
System B	0.14 ± 0.01	17.21 ± 0.02
System C	0.40 ± 0.01	17.14 ± 0.01
System C^†^	0.40 ± 0.06	17.11 ± 0.05
*Probe incorporated in the HPMC-based MBG* via *the HPMC/water mixture*		
System A	0.08 ± 0.01	17.35 ± 0.04
System B	0.15 ± 0.02	17.22 ± 0.03
System C	0.38 ± 0.03	17.11 ± 0.02

Systems A*, B*, and C*: mixtures of HPMC and water at ratios that correspond to the Systems A, B, and C, respectively; System C**: mixture of HPMC, water, and isooctane at ratios that correspond to System C; System C^†^: System C prepared with 0.2 M AOT microemulsion.

**Table 3:** *τ_R_*, *S*, and *A_N_* values of amphiphilic spin-probes 5-doxyl stearic acid (5-DSA) and 16-doxyl stearic acid (16-DSA) in AOT microemulsion (µE) and HPMC MBGs.

Amphiphilic Probes						
System	5 DSA			16 DSA		
	*τ_R_*, ns	*S*	*A_N_*, 10^−4^ T	*τ_R_*, ns	*S*	*A_N_*, 10^−4^ T
µE *w_o_* = 15	3.45 ± 0.10	0.29 ± 0.02	15.06 ± 0.03	0.07 ± 0.01	0.06 ± 0.01	14.39 ± 0.08
μE *w_o_* = 7.5	2.30 ± 0.08	0.14 ± 0.01	14.01 ± 0.05	0.09 ± 0.02	0.09 ± 0.01	14.26 ± 0.06
System A	6.02 ± 0.47	0.49 ± 0.03	15.08 ± 0.12	0.95 ± 0.01	0.05 ± 0.01	14.97 ± 0.02
System B	6.57 ± 0.05	0.55 ± 0.01	15.37 ± 0.05	1.39 ± 0.05	0.07 ± 0.01	14.85 ± 0.01
System C	6.96 ± 0.14	0.61 ± 0.03	15.69 ± 0.17	3.31 ± 0.04	0.17 ± 0.01	14.21 ± 0.14

**Table 4:** *τ_R_*, *S*, and *A_N_* values of free iodoacetamido-TEMPO in water and spin-labelled *C. rugosa* lipase in aqueous solution, AOT microemulsion, and Systems A and B.

Spin-Labelled Lipase from *Candida rugosa*—Iodoacetamide Tempo					
	*τ_R_*, ns			*S*	*A_N_*, 10^−4^T
Free spin label in water	0.06 ± 0.01			0.01 ± 0.01	17.48 ± 0.09
Spin-labelled *Candida rugosa* in aqueous solution					
Two components	Immobile	–	mobile		
	10.83 ns (44%)	–	1.66 ns (56%)	0.41 ± 0.03	17.23 ± 0.14
Spin-labelled *Candida rugosa* in AOT microemulsion, *w_o_* = 15					
Two components	Immobile	–	mobile		
	8.92 ns (20%)	–	1.78 ns (80%)	0.14 ± 0.06	16.00 ± 0.03
Spin-labelled *Candida rugosa* in HPMC-based MBGs					
Two components	Immobile	–	mobile		
System A	19.85 ns (45%)	–	3.16 ns (55%)	0.35 ± 0.04	16.48 ± 0.13
System B	21.87 ns (43%)	–	3.31 ns (57%)	0.39 ± 0.05	16.73 ± 0.08

**Table 5:** Correlation length, Porod, and Lorentz exponential.

Parameters	System C*	System C**	System C^‡^	System C^†^
Correlation length (Å)	44.6	44.1	66.9	71.6
Porod exponent (n)	4.5	4.5	4.8	5.1
Lorentzian exponent (m)	3.0	3.2	2.8	3.1

System C*: mixture of HPMC and water at ratios that correspond to System C; System C**: mixture of HPMC, water, and isooctane at ratios that correspond to System C; C^‡^: System C prepared with 0.05 M AOT microemulsion; C^†^: System C prepared with 0.2 M AOT microemulsion.

## Supplementary Materials

The following are available online at https://www.mdpi.com/2079-4991/10/11/2204/s1, Details of EPR analysis. Figure S1: Plots of rotational correlation time, *τ_R_*, vs. water content of Systems A, B, and C for the different spin probes used. Figure S2. Plots of hyperfine splitting constant, *A_Ν_*, vs. water content of MBG Systems A, B, and C for the different spin probes used. Figure S3. Spectra of 12-doxyl methyl stearate (12-DMS) in AOT microemulsion and in Systems A, B, C. Figure S4. Spectra of 5-doxyl decane (5-DD) in AOT microemulsion and Systems A, B, and C.
