# Supplementary material for: Structural Study of (Hydroxypropyl)Methyl Cellulose Microemulsion-Based Gels Used for Biocompatible Encapsulations
Source: Nanomaterials (Basel). 2020 Nov 5;10(11):2204. doi: 10.3390/nano10112204 (PMC7694351; doi:10.3390/nano10112204)
Supplement: Supplementary file 1 [file nanomaterials-10-02204-s001.pdf]

nanomaterials

MDPI

Article

Structural Study of (Hydroxypropyl)Methyl Cellulose Microemulsion-Based Gels Used for Biocompatible Encapsulations

Evdokia Vassiliadi [1,2], Evgenia Mitsou [1], Spyridon Avramiotis [1], Christos L. Chochos [1], Franz Pirolt [3], Martin Medebach [3], Otto Glatter [4], Aristotelis Xenakis [1] and Maria Zoumpanioti [1,*]

[1] Institute of Chemical Biology, National Hellenic Research Foundation, 48, Vassileos Constantinou Ave., 11635 Athens, Greece; evassiliadi@eie.gr (E.V.); emitsou@eie.gr (E.M.); spavramiotis@yahoo.com (S.A.); chochos@eie.gr (C.L.C.); arisx@eie.gr (A.X.)

[2] Laboratory of Biotechnology, Department of Biological Applications and Technologies, University of Ioannina, 45110 Ioannina, Greece

[3] Anton Paar GmbH, Anton Paar Straße 20, Graz 8054, Austria; franz.pirolt@anton-paar.com (F.P.); martin.medebach@anton-paar.com (M.M.)

[4] Institute of Inorganic Chemistry (6330), 8010 Stremayrgasse 9 Graz University of Technology, Graz, Austria; otto.glatter@uni-graz.at

* Correspondence: mariaz@eie.gr; Tel.: +30-210-727-3796

Supplementary Materials:

Details of EPR analysis

Calculation of the rotational correlation time, τ_R. The rotational correlation time, τ_R, is relevant to the spin probe's molecular motion. For the EPR time scale there are two regimes, namely, the fast-motion regime, $\tau_R < 3\text{x}10^{-9}$ s, and the slow-motion regime, $\tau_R > 3\text{x}10^{-9}$ s. The rotational correlation time values, τ_R, were calculated through computer simulations in all cases. For the fitting process the initial given values for the main magnetic parameters were: i) the tensor for the coupling between the electron spin and the magnetic field g_{xx}, g_{yy} and g_{zz}, values were 2.009, 2.006 and 2.003, respectively and they were allowed to vary ±0.001 and ii) the tensor for the coupling between the unpaired electron spin and the nuclear nitrogen spin (I_N = 1), A_{xx}, A_{yy} and A_{zz} values were 18, 18 and 93 MHz, respectively (or 6.4, 6.4 and $32.0\text{x}10^{-4}$ T) and they were allowed to vary ±20%. The Lorentzian and Gaussian contribution to the EPR line shape was also considered during the simulation procedure using the software parameters.

Calculation of the order parameter, S, and the hyperfine splitting constant, A_N. The *S* parameter value was obtained directly from the spectral characteristics. The order parameter *S* provides a measure of the spin probe's arrangement in a supramolecular assembly and varies from 0 to 1, with *S*=1 for the completely ordered state and *S*=0 for the completely random state [25] The *S* parameter is calculated as follows and as described elsewhere [26], [27]. A_N values are sensitive to the polarity of the immediate environment in which the spin probe resides and are increased when the polarity of the medium is increased, taking values from 17.50 x 10^{-4} T in a polar environment to 14.00 x 10^{-4} T in a non-polar environment [28], [29].

The *S* parameter is calculated from the following equation (2):

$$S = (A_{\|} - A_{\perp}) / [A_{zz} - (1/2)(A_{xx}+A_{yy})]\ (A_{N0}/A_N) \tag{2}$$

where $A_{\|}$ corresponds to the half-distance of the outer maximum hyperfine splitting, $2A_{max}$, and $A_{\perp}$ is calculated from the following equations (3) and (4):

$$A_{\perp} = A_{min} + 1.4(1 - S^{app}) \tag{3}$$

$$S^{app} = (A_{max} - A_{min})/[A_{zz} - (1/2)(A_{xx}+ A_{yy})] \quad (4)$$

where A_{min} is equal to the half-distance of the inner minimum hyperfine splitting. A_{N0} is the isotropic hyperfine splitting constant for the nitroxide molecule in the crystal state:

$$A_{N0} = (A_{xx} + A_{yy} + A_{zz})/3$$

$$A_N = (A_{\parallel} + 2A_{\perp})/3$$

The A_{xx}, A_{yy}, and A_{zz} are the single-crystal values of a nitroxide spin probe, equal to 6.3, 5.8, and 3.6 10^{-4}T, respectively.

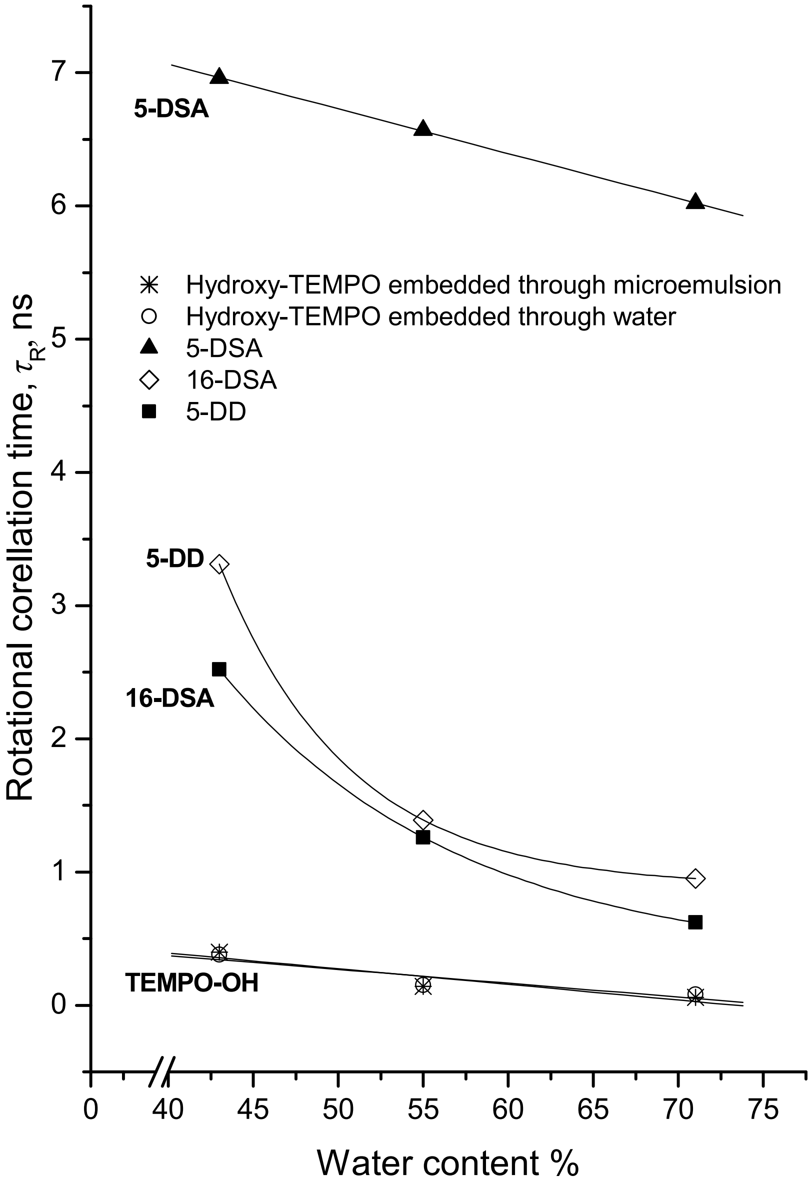

Figure S1. Plots of rotational correlation time, τ_R, vs water content of Systems A, B and C for the different spin probes used; (∗): Hydroxy-TEMPO embedded in HPMC MBGs through the microemulsion; (○): Hydroxy-TEMPO embedded in HPMC MGBs through the HPMC/water mixture; (▲): 5-DSA in HPMC MBGs; (◊): 16-DSA in HPMC MBGs; (■): 5-DD in HPMC MBGs.

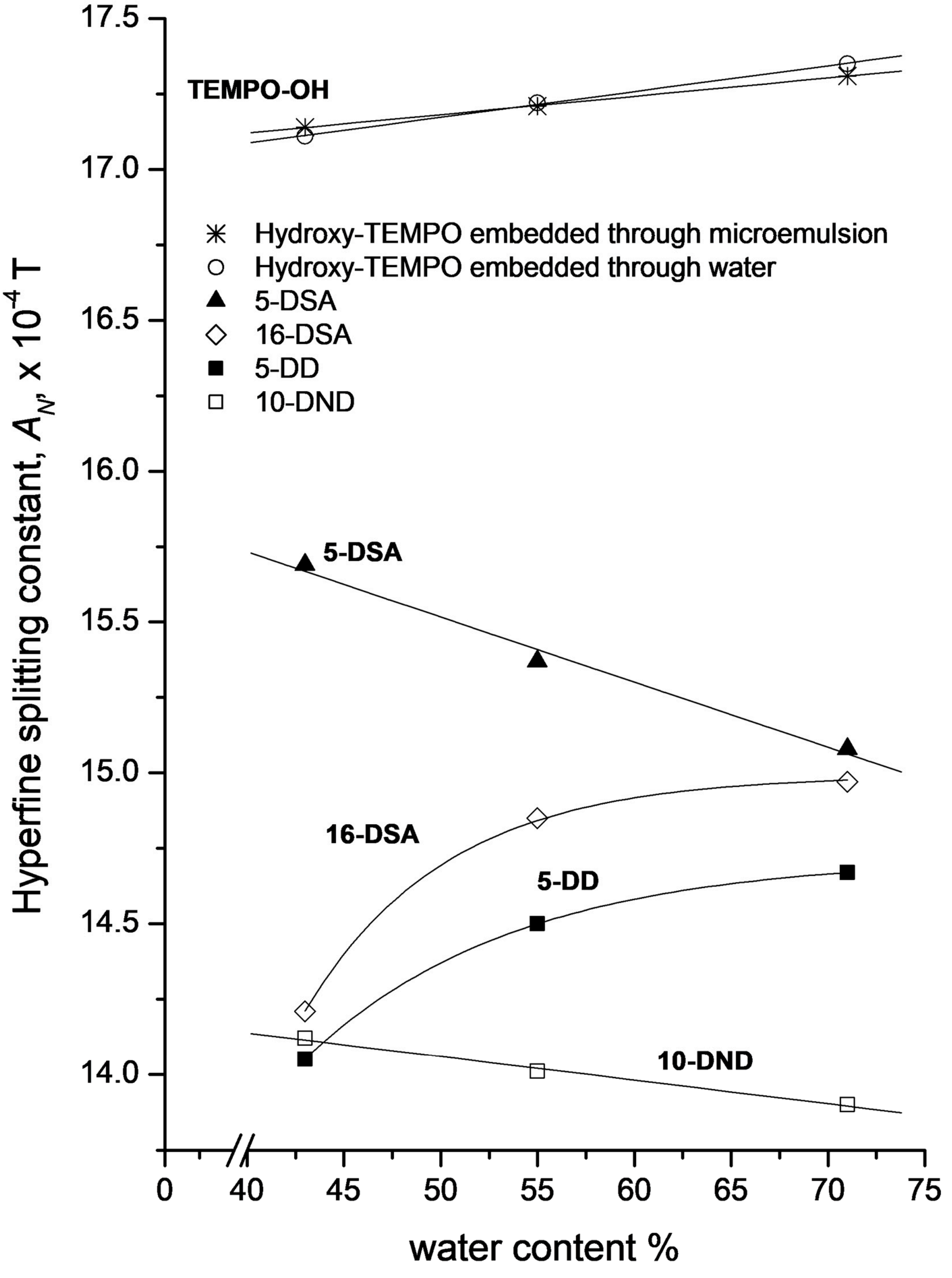

Figure S2. Plots of hyperfine splitting constant, A_N, vs water content of MBG Systems A, B and C for the different spin probes used. (∗): Hydroxy-TEMPO embedded in HPMC MBGs through the microemulsion; (○): Hydroxy-TEMPO embedded in MGBs through the HPMC/water mixture; (▲): 5-DSA in HPMC MBGs; (◊): 16-DSA in HPMC MBGs; (■): 5-DD in HPMC MBGs. (□):10-DND in HPMC MBGs.

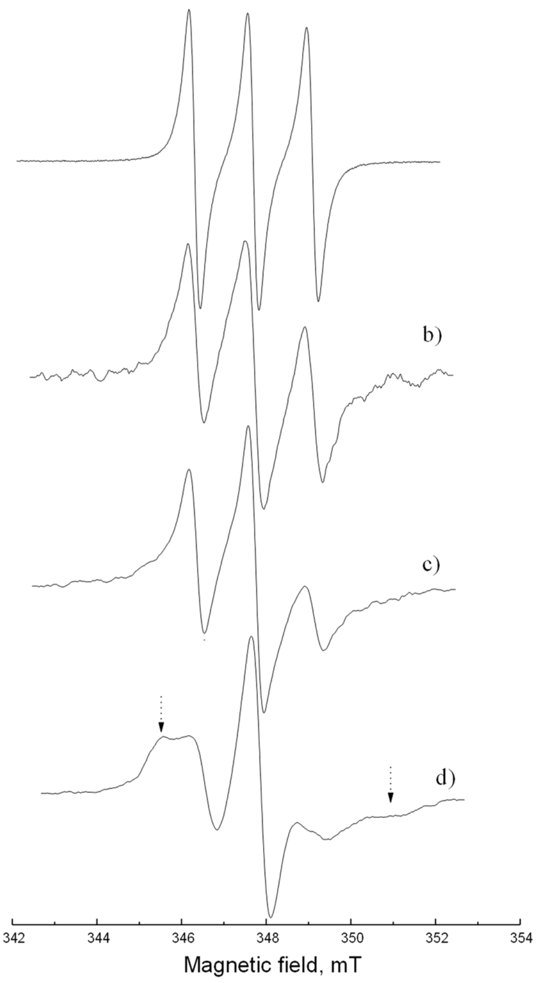

Figure S3. 12-doxyl methyl stearate (12-DMS) spectra in: (**a**) AOT microemulsion w_o=15;(**b**) System A; (**c**) System B; (**d**) System C; The arrows show increasing immobilization as the water decreased (broadening and splitting in the low field - splitting in the high field).

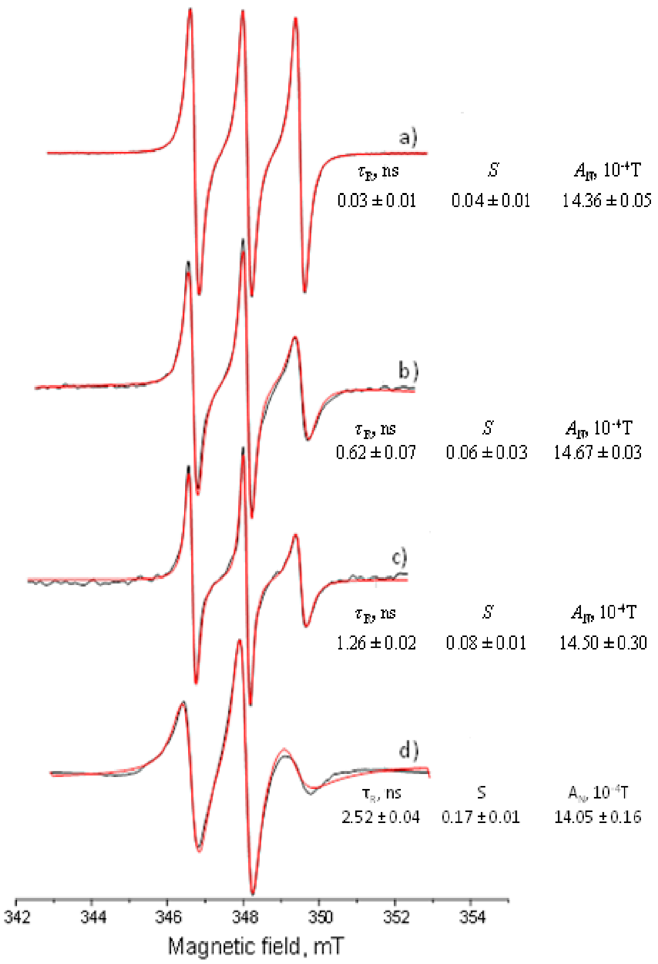

Figure S4. 5-doxyl decane (5-DD) spectra in: (**a**) AOT microemulsion w_o=15; (**b**) System A; (**c**) System B; (**d**) System C; black line experimental, red line simulation; for each spectrum are reported the τ_R, *S* and A_N values.

References

25. Griffith, O. H.; Jost, P. C. Lipid Spin Labels in Biological Membranes. In *Spin Labeling*; **1976**, Academic Press USA; *1*, pp. 453–523.
26. Papadimitriou, V.; Sotiroudis, T. G.; Xenakis, A. Olive Oil Microemulsions: Enzymatic Activities and Structural Characteristics. *Langmuir* **2007**, 23(4), 2071-2077.
27. Fanun, M.; Papadimitriou, V.; Xenakis, A. Characterization of Cephalexin Loaded Nonionic Microemulsions. *J. Colloid Interface Sci.* **2011**, 361(1), 115-121.
28. Knauer, B. R.; Napier, J. J. The Nitrogen Hyperfine Splitting Constant of the Nitroxide Functional Group as a Solvent Polarity Parameter. The Relative Importance for a Solvent Polarity Parameter of Its Being a Cybotactic Probe vs. Its Being a Model Process. *J. Am. Chem. Soc.* **1976**, 98, 4395-4400.
29. Marsh, D. Spin-Label EPR for Determining Polarity and Proticity in Biomolecular Assemblies: Transmembrane Profiles. *Appl. Magn. Reson.* ***2010***, *37*, 435-454.
